# Supplementary material for: Controlling fluid flow to improve cell seeding uniformity
Source: PLoS One. 2018 Nov 15;13(11):e0207211. doi: 10.1371/journal.pone.0207211 (PMC6237340; doi:10.1371/journal.pone.0207211)
Supplement: S1 File — This file contains all supplementary figures and information. (DOCX) [file pone.0207211.s002.docx]

# Supplementary


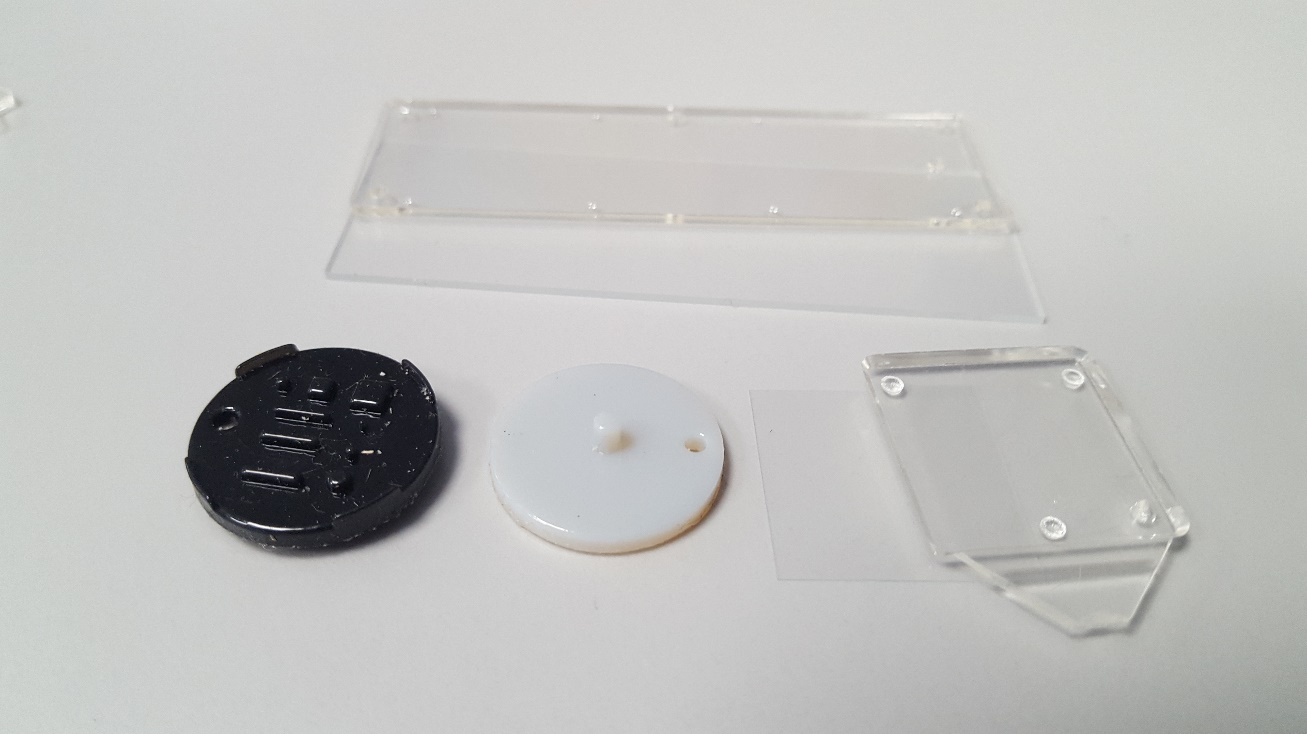


E

D

C

B

A


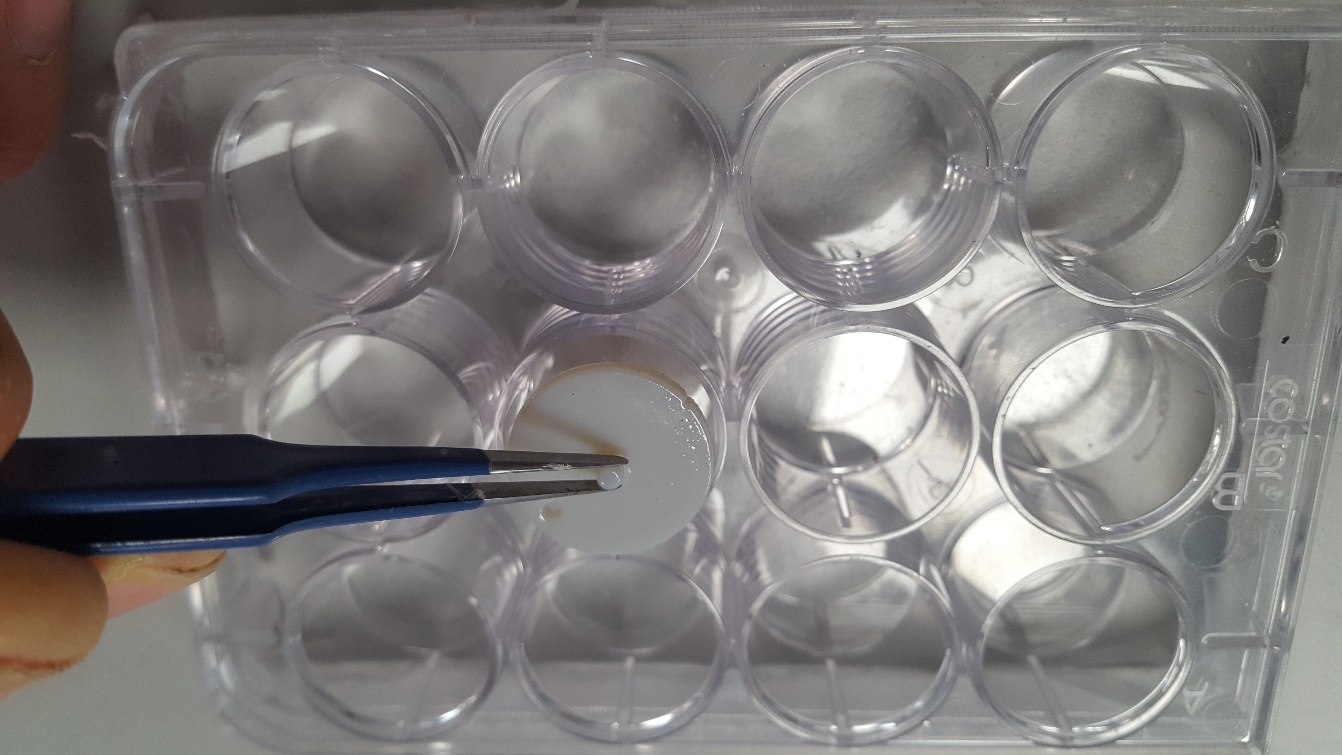


**Figure A: Various UCS geometries fabricated by injection moulding. and 3D printing.** Parts A and D are used to seed cells uniformly on microscope slides and 22mm coverslips respectively. Part B allows for millimetre scale patterning with a specific design in a 12 well dish, whilst part C is used for uniform seeding in a 12 well dish, as shown in E.


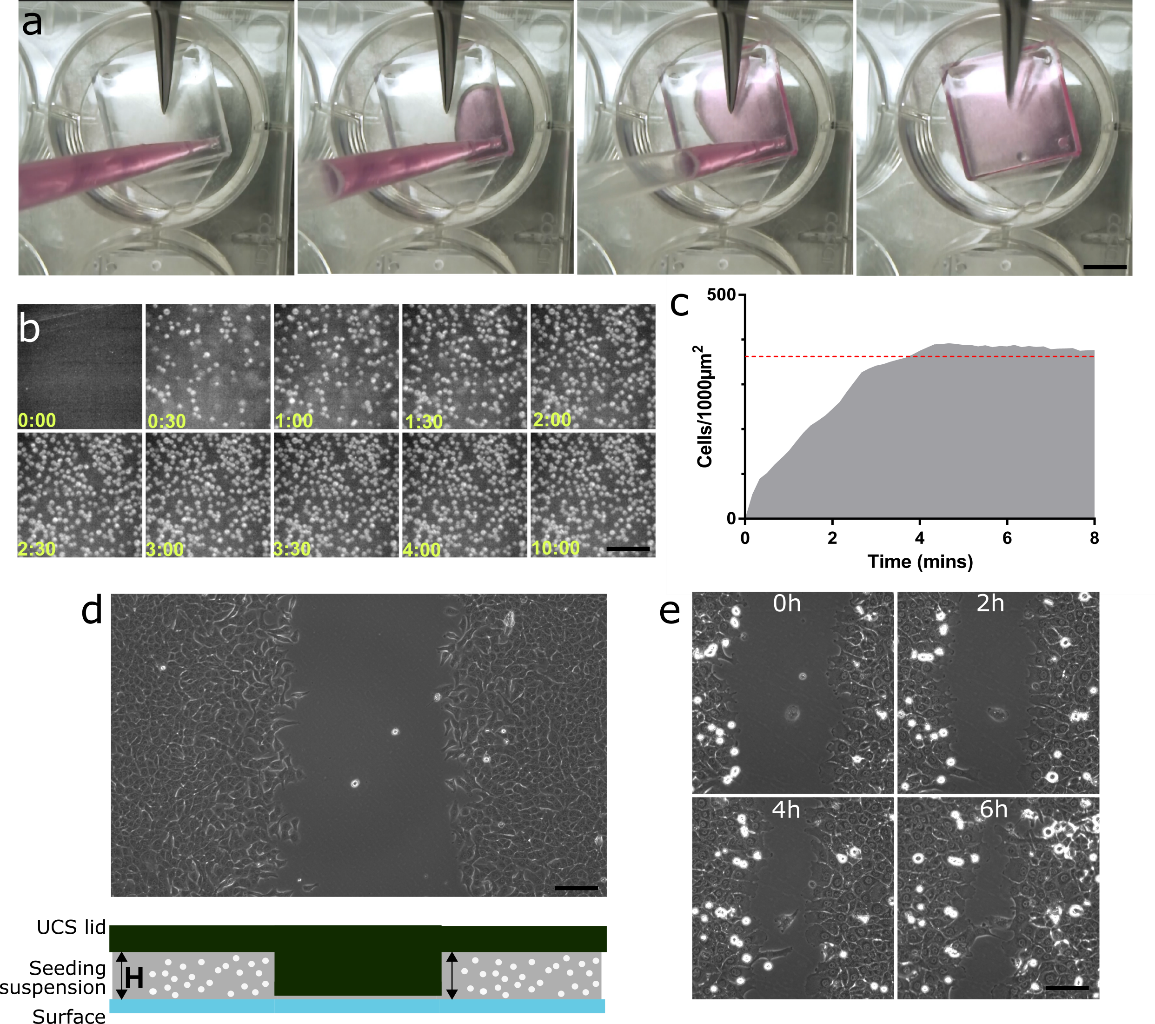


**Figure B: UCS device usage.** A) Cell suspension is added to the UCS device in under 3s. B) A modified internal reflection microscope was used to image cells as they settle to the culture surface over 10 mins. C) Target cell density is reached within 3 minutes at the target cell density (red dashed line).


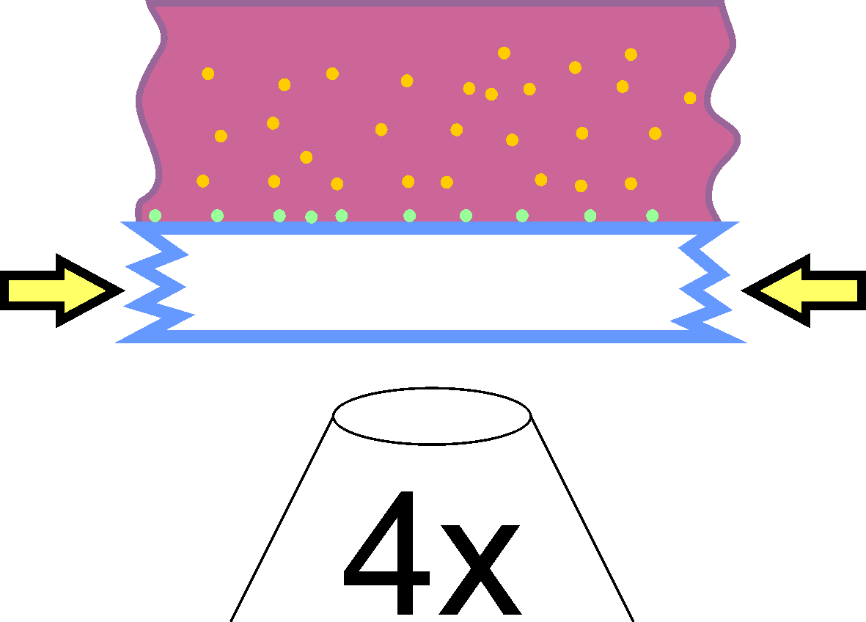


**Figure C: Schematic of modified internal reflection microscopy setup used to image cell sedimentation onto the culture surface in a wide field of view.** LED illumination was coupled into a 4mm thick glass plate from the side, and cells seeded using the UCS device above a low magnification (4x) objective. The only illumination source was the side coupled illumination. As cells settle on the surface, light is coupled from the glass plate and collected by a camera. As shown in S2 Figure.


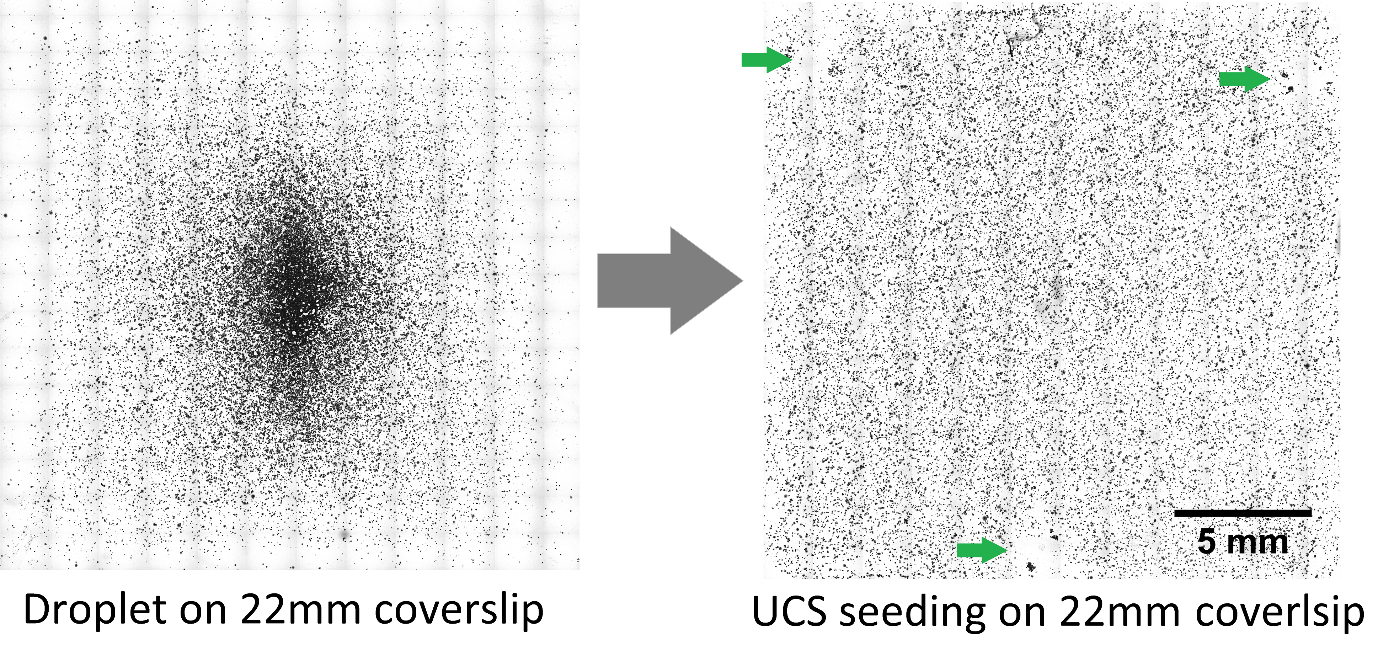


**Figure D: Correcting seeding variation on 22mm coverslip with the UCS device.** Standard practice to add a droplet of cell suspension to the prepared coverslip creates a dome of fluid which distributes cells in an uneven manner. The UCS device (right) corrects this by confining the seeding suspension at a uniform height across the coverslip. Defects still occur at the location of spacers on the UCS device (green arrows) however they represent a much lower fraction of the culture area.


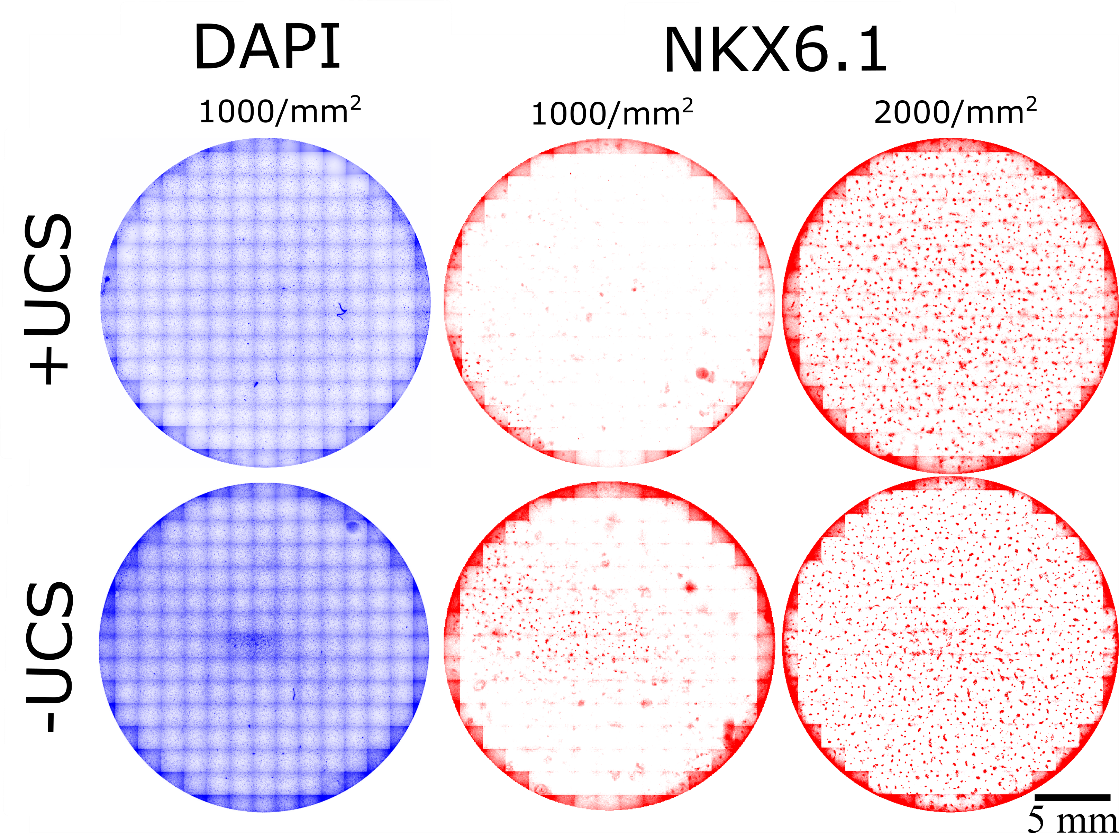


**Figure E: Uneven seeding at low densities may be beneficial.** hESCs re-seeded at high plating densities before differentiation towards PE. At low density aggregation of cells in the well centre when seeded without the UCS provides a region of sufficient local density to stimulate differentiation, as evidenced by large clusters of Nkx6.1 positive cells. When variation in cell density is corrected using the UCS device there are reduced numbers of differentiated clusters. At higher densities of 2000/mm^2^ there is a more uniform cluster morphology across the dish when cells are seeded using the UCS. DAPI = blue, PDX6.1 = red.

## UCS device for contact-free wound models

Given that the uniformity of the cell density is given by the height of the confined gap, it is possible to take advantage of this to pattern cells during the seeding phase. If a device has varying heights it would be possible to adjust the cell density locally and create contact-free wound healing models, Fig. S6. Generally wound models are either performed as a physical injury by scraping or as an adhesive barrier during the early stages of cell culture. However, both are likely to damage the surface and may disrupt chemical or biological coatings important for the wound study. To demonstrate this effect we 3D printed a number of different designs using different 3D printers. Depending on the type of printer, different degrees of quality can be obtained. Filament printing (FDM) is the most common type of printer in a research environment. This type of printer has limited edge resolution and will thus give rise to rounded corners and consequently less defined wounds. On the other hand, stereolithography (SLA) printers can achieve much high edge resolutions and consequently the definition of the wound is much sharper. As a consequence, when making UCS’ for wound healing experiments, the wound definition is limited in resolution.


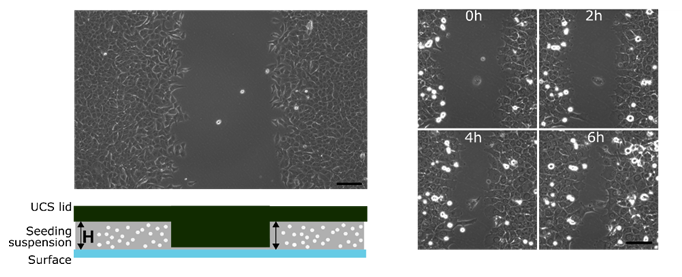


**Figure F: Contact-free gap closure assays created using the UCS.** Variation of the distance between the UCS lid and the culture surface can effectively exclude cells from a region (left). After removal of the device, gap closure can be measured.
